# Anesthetic effects and body weight changes associated with ketamine-xylazine-lidocaine administered to CD-1 mice

**DOI:** 10.1371/journal.pone.0184911

**Published:** 2017-09-14

**Authors:** Urshulaa Dholakia, Stuart C. Clark-Price, Stephanie C. J. Keating, Adam W. Stern

**Affiliations:** 1 Department of Veterinary Clinical Medicine, University of Illinois at Urbana-Champaign, Urbana, Illinois, United States of America; 2 Department of Pathobiology, University of Illinois at Urbana-Champaign, Urbana, Illinois, United States of America; National Center for Toxicological Research, UNITED STATES

## Abstract

Anesthesia for mice is commonly performed through the injection of parenteral agents via the intraperitoneal (IP) route. Variability in anesthetic sensitivities has been noted in mice resulting in inconsistencies in anesthetic depth and/or mortality. Anesthetic protocols that improve consistency and safety are needed. The objectives of this study were to assess the effects of intraperitoneal (IP) ketamine (95 mg/kg) and xylazine (7 mg/kg) alone or combined with lidocaine at 4, 8, or 16 mg/kg on time to loss (LRR) and return (RRR) of righting reflex, duration of immobilization and loss of pedal withdrawal response (PWR), body weight and histopathology in CD-1 mice. In a prospective, randomized trial, 36 male CD-1 mice, 4–6 weeks of age were randomly assigned to 5 groups: saline (SA, n = 4); ketamine-xylazine (KX, n = 8); ketamine-xylazine-lidocaine 4 mg/kg (KXL4, n = 8); ketamine-xylazine-lidocaine 8 mg/kg (KXL8, n = 8); ketamine-xylazine-lidocaine 16 mg/kg (KXL16, n = 8). Two mice in each group were euthanized at day 2 post-injection and the remaining mice were euthanized at day 11 post-injection. After IP injection, LRR and RRR, duration of immobilization and loss of PWR, body weight and histopathology were evaluated. LRR occurred sooner in mice receiving KXL16 compared with KX, with median (range) times of 78 (62–104) and 107 (91–298) seconds, respectively. Loss of PWR occurred in 1, 5, 4, 6 mice for groups KX, KXL4, KXL8, and KXL16 respectively. Median (range) duration of absent PWR was longer in mice receiving KXL16 at 13 (0–30) minutes, compared to KX at 0 (0–9) minutes. Duration of immobilization and RRR were not different between groups. Weight loss occurred 2 days following anesthesia but was not different between groups. Weight gain was significantly greater in all lidocaine groups 11 days post-injection compared to KX. No mortality or histopathologic abnormalities were observed in any group. Lidocaine administered with ketamine and xylazine shortens the onset of anesthesia in mice and improves anesthetic depth without prolonging recovery time.

## Introduction

Anesthesia for small rodents is commonly performed through the injection of one, or a mixture of parenteral agents [1–3]. The intraperitoneal (IP) route of administration is often utilized for laboratory mice when there is an inability to use inhalant anesthesia for research-specific requirements, lack of inhalational anesthesia equipment, or due to technical limitations of intravenous or intramuscular injections in small species [4]. Ketamine is frequently combined with xylazine to induce and maintain anesthesia for a variety of procedures in laboratory mice [5]. However, a great deal of variability in anesthetic sensitivities has been noted based on strain, sex, and age, such that inconsistencies in anesthetic depth and/or mortality among individual rodents remains a concern [6–8]. Using balanced anesthesia to reduce total doses of each individual agent in order to minimize adverse effects, while providing sufficient analgesia, is especially important for small rodents [4]. Numerous studies have shown advantages of the addition of sedative and/or analgesic medications to improve the quality or duration of anesthesia in rodents [1,4,6,9].

Systemically administered lidocaine has been used as an adjunct to balanced anesthesia protocols in several species, including rodents, rabbits, pigs, and humans [10–14]. Lidocaine acts primarily upon voltage-gated sodium channels to inhibit generation and propagation of action potentials in electrically active tissues such as nerves, muscle, and cardiac muscle. Other postulated mechanisms of action include the induction of acetylcholine release in the CSF, inhibition of glycine receptors, and release of endogenous opioids in human and veterinary patients [10,15]. Lidocaine reduces isoflurane requirements for surgery in dogs and horses [16,17], contributes to multimodal analgesia in combination with other agents for the treatment of postoperative pain in dogs, cats, goats, horses, calves, rats, and humans [15], and has anti-inflammatory in humans, horses, and dogs [10,15,18–20]. Local anesthetics such as lidocaine have demonstrated in vitro antimicrobial effects on bacterial isolates from humans and horses [21,22]. In addition, lidocaine is readily available as a sterile, pharmaceutical-grade product, inexpensive, and not subject to state or DEA controlled substance regulation. Intravenous infusion of lidocaine to C57BL/6 mice has been shown to reduce ketamine and medetomidine maintenance anesthetic drug requirements [13]. In rats, the inclusion of lidocaine to intraperitoneally-administered pentobarbital has been shown to reduce pain behavior in comparison to injection of pentobarbital alone, and reduce c-fos expression, an indicator of stress, in spinal cord neurons [23]. Despite studies demonstrating the benefits of systemically administered lidocaine, there is limited literature evaluating clinical use of lidocaine for general anesthetic protocols in mice. The current study was performed to determine how the inclusion of lidocaine in ketamine/xylazine-anesthetized mice would affect the duration of anesthesia, response to noxious stimulation, changes in post-anesthetic body weight and histopathologic findings following intraperitoneal injection in CD-1 mice. The hypothesis tested was that the addition of lidocaine to an IP injection of ketamine and xylazine in mice would result in a more rapid induction and a deeper plane of anesthesia.

## Materials and methods

### Animals

Thirty six, male CD-1(ICR) mice (age 4–6 wk) were acquired from Charles River Laboratories (Wilmington, MA USA). Mice were housed in compatible groups within individually ventilated microisolator cages (Microvent, Allentown Caging Equipment), provided with free choice standard rodent diet (Teklad Rodent Diet 8604, Envigo), bottled tap water, autoclaved corncob bedding, and cotton nesting material. All mice were weighed prior to the experiment, and then daily throughout the study period. This study was approved by the University of Illinois Urbana-Champaign Institutional Animal Care and Use Committee (protocol #15195), and mice were housed within the university’s AAALAC-accredited animal facility. Husbandry, health monitoring, and experimental procedures were in compliance with the recommendations of the *Guide for the Care and Use of Laboratory Animals* (NRC, 2011).

### Experimental groups

Mice were randomly assigned by use of a random number generator (random.org) to one of 5 groups: saline control (SA, n = 4); ketamine-xylazine (KX, n = 8); ketamine-xylazine-lidocaine 4mg/kg (KXL4, n = 8); ketamine-xylazine-lidocaine 8mg/kg (KXL8, n = 8); ketamine-xylazine-lidocaine 16mg/kg (KXL16, n = 8). Two mice in each group were euthanized at day 2 post-injection and the remaining mice were euthanized at day 11 post-injection. Animals were euthanized by carbon dioxide inhalation according to *American Veterinary Medical Association Guidelines for the Euthanasia of Animals*, 2013. Cervical dislocation was subsequently performed to ensure death, after respiratory arrest. All mice were submitted for gross necropsy and histopathology of thoracic and abdominal organs. A minimum sample size of 7 mice per experimental group was determined to be necessary to detect a difference between groups of a 60 second decrease in time to LRR with a sigma of 60, and alpha of 0.05, and a power set to 0.80. The sigma value was obtained from a previous study evaluating xylazine-ketamine anesthesia in mice [24]. To ensure statistical relevancy and to account for the potential for error during the study, the number of mice was increase to 8 per experimental group.

### Anesthetic dilution and administration

All anesthetic agents were USP grade, commercially available formulations. Drugs were diluted using sterile 0.9% sodium chloride for injection (Hospira), and stored separately in sterile glass vials at room temperature for no more than 7 days. Ketamine hydrochloride (Zetamine, VetOne) was diluted to a concentration of 10mg/mL; xylazine hydrochloride (Anased, Akorn Inc) was diluted to a concentration of 2mg/mL; and lidocaine hydrochloride 2% (Hospira) was diluted to 2mg/mL (for groups KXL4 and KXL8) and to 4mg/mL for administration to group KXL16. The maximum dose of 16 mg/kg of lidocaine was selected as it remains below toxic doses in mice [25,26]. Control animals were administered 0.5 mL of sterile 0.9% sodium chloride. Ketamine (95 mg/kg) and xylazine (7 mg/kg) were administered to all experimental groups. Ketamine and xylazine doses were determined based on the authors’ previous experience, and fall within published reference ranges recommended for mice. Lidocaine was administered according to experimental group. Anesthetics were combined into a single syringe for intraperitoneal administration just prior to dosing. Mice were manually restrained and injected with similar technique, using a 1mL tuberculin syringe with 25g needle into the right lower quadrant of the abdomen. Total volumes for IP injection were no greater than 20μL/g.

### Anesthesia and data collection

Mice were weighed individually immediately prior to dosing. Following IP injection, mice were placed into a clean, bedded cage. Simultaneously, a stopwatch was initiated to record the time to loss of righting reflex (LRR), and all subsequent time measures. Upon LRR, mice were removed from the cage and placed in dorsal recumbency on a disposable absorbent pad, on top of a thermostatically-controlled pad (Small Animal Heated Pad, H&K) for thermal support. Eyes were lubricated using artificial tears ointment (Rugby Laboratories). Pedal withdrawal response (PWR) was checked at one minute intervals, using digital pressure on the hind toes. The same investigator (UD) performed all PWR to maintain consistency. Digital pressure was chosen over mechanical forceps in order to avoid traumatic injury to the toes, with purposeful withdrawal of the limb considered a positive response. The time to return of righting reflex (RRR) was recorded for each mouse. Once alert and ambulatory, mice were returned to their home cage. All procedures took place between 0900 and 1200, to control for circadian variation.

### Samples

At the designated experimental time points, mice were euthanized and immediately submitted to the Veterinary Diagnostic Laboratory, University of Illinois at Urbana-Champaign for blinded necropsy examination and histopathology. Tissue specimens collected were preserved in 10% buffered formalin with fixation times of approximately 24–72 hours prior to processing. Samples were embedded in paraffin and sections cut at 3 μm, and stained with hematoxylin and eosin (HE).

### Statistics

Data were analyzed for normality with a Kolmogorov-Smirnov test. Times to loss and return of righting reflex, and duration of loss of PWR were analyzed with a Kruskall-Wallis test and a post hoc Dunn’s multiple comparisons test when significant. A Cochran-Armitage test for trend was used to analyze the loss of PWR. Percent change in body weights 11 days post-injection was calculated in all surviving mice as [(weight on day 11—weight on day zero)/weight on day zero] X 100 and were analyzed with ANOVA and a post hoc Tukey-Kramer multiple comparisons test and reported as mean ± SD. A commercial statistical program was utilized for all analysis (InStat®, GraphPad Software, Inc. La Jolla, CA, USA). A P value of <0.05 was considered significant.

## Results

All mice recovered from anesthetic trials in all groups, with no mortality or other adverse events observed. Times for LRR, RRR and PWR for each group are summarized in Table 1.Mice in all anesthetic groups lost righting reflex following IP injection. Time to LRR for group KXL16 was significantly shorter than the KX group (P = 0.002). There was no statistical difference between times to LRR between any other groups. All mice in all anesthetic groups regained righting reflex by two hours after IP injection. There was no statistical difference between times to RRR between groups. Loss of PWR occurred in only 1 of 8 mice in the KX group (12.5%); in 5 of 8 mice in the KXL4 group (62.5%); in 4 of 8 mice in the KXL8 group (50%); and in 6 of 8 mice in the KXL16 group (75%). There was a significant linear trend toward loss of PWR (P = 0.027) as the dose of lidocaine was increased. The total duration of loss of PWR in group KXL16 was significantly greater than the KX group (P = 0.027). There was no statistical difference in duration of loss of PWR between any other groups. Additionally, only mice in group KXL16 maintained a consistent loss of PWR over consecutive minutes, whereas mice in other groups demonstrated intermittent loss and return of PWR.

**Table 1:** Median (range) summary data of mice after intraperitioneal injection with ketamine/xylazine ± lidocaine.

	KX	KXL4	KXL8	KXL16
LRR (seconds)	107 (91–298)	101 (85–162)	102 (88–141)	78 (62–104)^a^
RRR (minutes)	47 (36–67)	53 (31–68)	41 (32–54)	42 (37–49)
TDI (minutes)	39 (30–53)	41 (26–47)	27 (21–45)	32 (28–38)
Loss of PWR (minutes)	9 (9)	6 (1–13)	11(1–17)	16.50(11–30)^a^
Number of mice that lost PWR in group (%)	1/8 (12.5)	5/8 (62.5)	4/8 (50)	6/8 (75)

KX, ketamine (95 mg/kg)-xylazine (7 mg/kg), KXL4, ketamine-xylazine-lidocaine (4 mg/kg), KXL8, ketamine-xylazine-lidocaine (8 mg/kg), KXL16, ketamine-xylazine-lidocaine (16 mg/kg), LRR, loss of righting reflex, RRR, return of righting reflex, TDI, total duration of immobilization, PWR, pedal withdrawal response.

^a^ Indicates significant difference (P < 0.05) from KX.

Body weight data are summarized in Table 2. Pre-injection (baseline) body weight was not significantly different between control or anesthetic groups, and all anesthetic groups demonstrated a loss of body weight during the first 2 days post-injection. There was no significant difference in the percent of weight loss experienced in the first 2 days between anesthetic groups. There were 6 mice remaining for each anesthetic group on day 11 that were included in the day 11 body weight analysis. However, only 2 mice remained alive in the control group at that time point. Due to the low number of animals, the control group was not included in the body weight statistical analysis. Body weight 11 days post-injection increased 2.2 ± 2.9% in group KX, 7.4 ± 4.1% in group KXL4, 7.3 ± 2.0% in group KXL8, and 11.9 ± 2.0% in group KXL16 from baseline. Mice in group KXL4 (p<0.05), KXL8 (p<0.05), and KXL16 (p<0.001) had a significantly greater increase in post-anesthetic body weight compared to mice in group KX.

**Table 2:** Mean ± SD body weight change after intraperitioneal injection with ketamine/xylazine ± lidocaine in mice.

	KX	KXL4	KXL8	KXL16
Baseline body weight, g	34.9 ± 2.2	34.5 ± 4.3	32.4 ± 1.5	32.0 ± 2.0
Weight (%) change, 2 days post	-1.6 ± 1.6	-0.5 ± 1.7	-1.3 ± 1.3	-0.8 ± 2.1
Weight (%) change, 11 days post	2.2 ± 2.9	7.4 ± 4.1^a^	7.3 ± 2.0^a^	11.9 ± 2.0^b^

KX, ketamine (95 mg/kg)-xylazine (7 mg/kg), KXL4, ketamine-xylazine-lidocaine (4 mg/kg), KXL8, ketamine-xylazine-lidocaine (8 mg/kg), KXL16, ketamine-xylazine-lidocaine (16 mg/kg).

^a^ Indicates significant difference (P < 0.05) from group KX.

^b^ Indicates significant difference (P <0.001) from group KX.

### Necropsy

All mice were in good overall health and body condition. No gross abnormalities were observed in any of the day 2 post-injection mice or the day 11 post-injection mice.

### Histopathology

The lung, heart, liver, kidney, spleen, small intestine and mesentery, and abdominal body wall were examined from all mice. All mice had a minimal to mild vacuolar hepatopathy (consistent with glycogen) and was considered an incidental finding. Two mice in the KXL16 group (one 2-day mouse and one 11-day mouse) had small numbers of cytoplasmic vacuoles within the cardiac interstitium and was not considered to be a clinically relevant finding. No other cardiac changes including inflammation, fibrosis or necrosis were observed in any sections of heart examined. Mesothelial cells lining the abdominal body wall were non-reactive. No abnormalities were found on any other tissue sample from all mice. Tissue from the central nervous system of mice was not examined.

## Discussion

To the authors’ knowledge, this study represents the first evaluation of intraperitoneal lidocaine to supplement ketamine-xylazine anesthesia in mice. The results are supportive of earlier studies demonstrating beneficial effects of systemically administered lidocaine as an adjunct to general anesthesia. Lidocaine has previously been demonstrated to reduce the minimum alveolar concentration of inhalant anesthetics in rabbits [12], dogs [16,27], horses [17], and to reduce opioid requirements in humans [28]. Lidocaine is also used as a co-induction agent in some species. Intravenous administration of 2 mg/kg lidocaine prior to induction with propofol has been shown to decrease consciousness [29], and to minimize and/or eliminate pain associated with propofol injection [30]. Inclusion of lidocaine with pentobarbital administered intraperitoneally to male Wistar Hanover GALAN rats reduced Fos-like immunoreactivity of spinal neurons by one-third in comparison to pentobarbital alone [23]. That study concluded that the addition of lidocaine to a moderately painful, alkaline solution (pentobarbital) reduced nociceptive inputs from injection, thereby serving as a refinement for animal welfare.

In consideration of these previous studies, we hypothesized that the addition of lidocaine to other injectable anesthetic solutions used in mice, such as ketamine/xylazine, would demonstrate similar beneficial clinical characteristics. All mice administered lidocaine-supplemented anesthesia experienced a rapid and smooth induction following IP injection, with a zero failure rate, as all mice lost righting reflex and became immobilized. Additionally, LRR was significantly faster in mice administered the KXL16 combination than KX alone. The admixture of lidocaine with ketamine hydrochloride, an acidic solution supplied at pH of 3.5–5.5, into the same syringe may have alleviated pain associated with IP injection in our mice, resulting in less excitement and/or distress. Lidocaine is known to have a rapid onset of action, less than two minutes [15]. Alternatively, lidocaine may have had a more direct, centrally-acting effect to enhance the actions of ketamine and xylazine for more rapid induction of unconsciousness [10]. The higher lidocaine dose required to elicit this effect approaches a similar range to that used in a prior study evaluating paw-licking responses to formalin in male Swiss albino mice [31]. In this experiment, IP lidocaine doses of 20–30 mg/kg were required to produce significant antinociceptive effects. The dose ranges used in the current study were initially based upon those used in another study in which lidocaine was given as a continuous intravenous infusion at 2, 4, and 8 mg/kg/hr, with no hemodynamic issues noted to occur in the mice [13]. The maximum dose of 16 mg/kg of lidocaine was designed to remain below other published values for CNS toxicity [25], and cardiotoxicity in mice [26].

In contrast to studies investigating other anesthetic adjunctive agents, the duration of anesthesia for male CD-1 mice in the current study was not dose-dependent with lidocaine; even at the highest dose used, the duration of immobilization and time to RRR remained comparable to that of ketamine/xylazine used alone. Previous adjunctive agents attempted in mice include acepromazine [32,33], midazolam [34], and numerous permutations of other alpha-2 agonists, dissociatives, and opioids [2,6,35]. In most cases, the addition of long-acting drugs resulted in increased duration of immobilization, which potentially delays recovery from hypothermia, a significant factor contributing to rodent mortality. In particular, use of acepromazine as an adjunct to ketamine/medetomidine in female BALB/c mice was shown to significantly prolong the time to recovery [36]. The relatively short duration of action of lidocaine, with a half-life of 0.5–2 hours following intravenous administration [15], may have contributed to its rapid elimination and facilitated a more rapid recovery compared to other anesthetic adjuncts.

Similar to prior studies and reviews of injectable anesthesia protocols for mice, great variability in the response to external stimuli of individual mice to ketamine-xylazine and lidocaine supplemented groups was observed [6–8]. This may be related to methods of evaluation, as limb movements do not necessarily correspond to conscious perception [37–39]; however, many institutions utilize loss of PWR as a sign of adequate surgical depth [8,36]. Lidocaine has previously been evaluated for stability as a diluted solution for up to 14 days, and no incompatibilities have been noted [40]. Therefore, it is unlikely that mixture of the drug with saline, ketamine, or xylazine contributed to the variability as a result of issues with stability or potency. Instead, it may be attributed to differing rates of IP absorption, or the nature of individual animals. Despite the observed variability, mice in group KXL16 showed a consistent and statistically significant increase in duration of loss of PWR compared to group KX. Additionally, there was a dose-dependent effect of lidocaine on loss of PWR. It appears that as the dose of lidocaine is increased, mice are more likely to lose PWR. However, larger groups, cross-over designed studies, different strains of mice, and other combinations and doses of lidocaine should be studied to further define doses and techniques that provide more consistent results.

Despite the observed variability, the loss of PWR was expected based on the previously reported properties of lidocaine. Specifically, intravenous lidocaine has been noted to decrease laryngeal reflexes at intubation in dogs [41,42], and children [43]. In addition, lidocaine continuous-rate infusion confers an anesthetic-sparing effect in mice [13], and allows for dose reduction of inhalants and opioids through perioperative analgesic effects [11,27].

In rodents, post anesthetic weight loss has been noted to persist for up to 48 hours, and is nonspecific with regard to use of inhalant or parenteral agents [44–47]. Within the present study, all mice other than the control group lost weight during the first 2 days following anesthesia. Mice did not demonstrate other clinical signs of distress, and were otherwise active, hydrated, and apparently healthy. Similarly, in a study of Long-Evans and Sprague-Dawley rats, animals were noted to lose 2.5% ± 0.56 of their body weight 24 hours following anesthesia alone using ketamine/xylazine/acepromazine [45]. Acute loss of body weight, and/or body condition, is often used as an indicator of pain or distress, and as an objective measure for humane endpoints for laboratory animals. Few studies clearly differentiate weight loss effects between anesthetic protocols and other contributing factors [48]. Therefore, the mice in this study were monitored over the course of 11 days following recovery to assess for influences of the anesthetic protocol on longer-term body weight and overall health. The inclusion of lidocaine into the anesthetic protocol did not affect weight loss in the first 2 days following anesthesia at any of the doses administered. However, mice in all anesthetic groups eventually regained weight by day 11 post-injection, and most interestingly, all lidocaine-supplemented groups showed significantly higher weight gains at 11 days. Mice in group KXL16 had the greatest weight gain at day 11 post-injection and thus the greatest difference compared to mice in group KX. The physiologic mechanism for greater weight gain is not clear, as a single-bolus of lidocaine administered at the time of anesthesia would be unlikely to remain clinically relevant in terms of serum levels for 11 days. It is possible that lidocaine promoted a faster return to normal feeding behaviors and/or normal gastrointestinal function after recovery from immobilization. All groups of mice were fed a similar diet, ad libitum. However, feed and water consumption of each group was not quantified, therefore it is unknown if the lidocaine groups returned to feeding behaviors sooner. Reduction of inflammation or irritation of the gastrointestinal tract from the administration of ketamine and/or xylazine by the inclusion of lidocaine was considered as a possible cause of the increased weight gain in the lidocaine groups. However, necropsy of 2-day post and 11-day post-injection mice did not reveal any gross or histologic evidence of gastrointestinal abnormalities in any mice of all groups. Large, retrospective studies in the human literature are equivocal with regard to the benefits of systemic lidocaine for promoting faster return to normal gastrointestinal function and reducing hospital stay [49]. Other reviews suggest greatly improved patient comfort, and reduced time to hospital discharge [11,49]. Post-operative ileus is one complication following general anesthesia in horses, and can often be effectively managed using intravenous lidocaine prophylactically [50]. Further investigation in regard to the effects of lidocaine on food and water intake of mice following anesthesia is warranted.

A weakness of this study was the lack of blinding of the investigator assessing PWR. This could have led to unintentional bias in the assessment of PWR. However, the other parameters assessed (LRR, RRR, and body weight) are objective measures and likely not affected by lack of blinding. Additionally, because of randomization of treatments, on any given day of assessment, the order of mice receiving any of the four treatments was random and therefore it would not have been possible to assess for any trend in response until the study was completed and statistical analysis was performed minimizing bias. With that said, future studies will utilize blinding during assessment. Gross and histopathology was performed without knowledge of treatment groups.

## Conclusions

The administration of lidocaine concurrently with ketamine and xylazine resulted in a faster onset of general anesthesia, improves anesthetic depth, and does not extend the duration of time to recovery of CD-1 mice. Additionally, a significant increase in time of reduced PWR for the KXL16 mice was noted. Optimum effective doses for other strains of mice, older animals or those with co-existing conditions may vary from the present study, and should be determined separately based on initial pilot studies. Further studies investigating the use of intraperitoneal lidocaine in mice is warranted.

## Supporting information

S1 File Raw data. Raw data of mice after intraperitioneal injection with ketamine/xylazine ± lidocaine.
(XLSX)
